# Spatial variation in western corn rootworm (Coleoptera: Chrysomelidae) susceptibility to Cry3 toxins in Nebraska

**DOI:** 10.1371/journal.pone.0208266

**Published:** 2018-11-29

**Authors:** Jordan D. Reinders, Brianna D. Hitt, Walter W. Stroup, B. Wade French, Lance J. Meinke

**Affiliations:** 1 Department of Entomology, University of Nebraska, Lincoln, Nebraska, United States of America; 2 Department of Statistics, University of Nebraska, Lincoln, Nebraska, United States of America; 3 North Central Agricultural Research Laboratory, United States Department of Agriculture–Agricultural Research Service, Brookings, South Dakota, United States of America; Chinese Academy of Agricultural Sciences, CHINA

## Abstract

Repeated use of field corn (*Zea mays* L.) hybrids expressing the Cry3Bb1 and mCry3A traits in Nebraska has selected for field-evolved resistance in some western corn rootworm (WCR; *Diabrotica virgifera virgifera* LeConte) populations. Therefore, this study was conducted to characterize spatial variation in local WCR susceptibility to Cry3Bb1 and mCry3A traits in Keith and Buffalo counties, Nebraska, and determine the relationship between past management practices and current WCR susceptibility. Adult WCR populations were collected from sampling grids during 2015 and 2016 and single-plant larval bioassays conducted with F_1_ progeny documented significant variation in WCR susceptibility to Cry3Bb1 and mCry3A on different spatial scales in both sampling grids. At the local level, results revealed that neighboring cornfields may support WCR populations with very different susceptibility levels, indicating that gene flow of resistant alleles from high trait survival sites is not inundating large areas. A field history index, comprised of additive and weighted variables including past WCR management tactics and agronomic practices, was developed to quantify relative selection pressure in individual fields. The field history index-Cry3 trait survivorship relationship from year 1 data was highly predictive of year 2 Cry3 trait survivorship when year 2 field history indices were inserted into the year 1 base model. Sensitivity analyses indicated years of trait use and associated selection pressure at the local level were the key drivers of WCR susceptibility to Cry3 traits in this system. Retrospective case histories from this study will inform development of optimal resistance management programs and increase understanding of plant-insect interactions that may occur when transgenic corn is deployed in the landscape. Results from this study also support current recommendations to slow or mitigate the evolution of resistance by using a multi-tactic approach to manage WCR densities in individual fields within an integrated pest management framework.

## Introduction

The western corn rootworm (WCR), *Diabrotica virgifera virgifera* LeConte, is one of the most serious pests of field corn (*Zea mays* L.) in the United States Corn Belt. North American *Diabrotica* species are responsible for upwards of $1 billion annually in control costs and yield losses [1–4]. Root feeding by WCR larvae can significantly decrease plant growth and biomass by reducing water and nutrient uptake under moderate to high population densities [5–7]. The impact of this WCR-corn association on yield is dictated by the interaction of WCR density with hybrid genetics and environmental conditions [8,9]. For every node of root injury [10], a 15–17% yield reduction is possible [4,11]. Adult feeding on corn silks under some conditions can significantly reduce pollination, also facilitating reduced grain yield [12,13].

Various tactics are used to manage WCR injury in field corn. Because of the strong affinity of WCR to oviposit in corn [14,15], annual rotation from corn to a crop that is not a WCR host has been a good cultural management approach. This management approach does not fit all farm business models, especially where high volumes of corn are needed for livestock or ethanol production or the economics of corn production is better than available rotational crops. Therefore, continuous corn production (≥ 2 successive years of corn in one field) can be common, especially under irrigation in the western U.S. Corn Belt [16,17]. Foliar- or soil-applied insecticides targeting WCR adult or larval stages, respectively, have been used in continuous corn since the late 1940s for WCR control [18–22]. The WCR has been highly adaptive to these management tactics as field-evolved resistance to crop rotation [23] and four classes of insecticides [22,24,25] has been reported.

In 2003, transgenic corn expressing the insecticidal protein Cry3Bb1 derived from the soil bacterium *Bacillus thuringiensis* Berliner (Bt) was commercially introduced as a WCR management tactic [26,27]. This was followed by the registration of Cry34/35Ab1 in 2005 [28], mCry3A in 2006 [29], and eCry3.1Ab in 2012 [30]. The efficacy, increased farmer safety, and convenience of transgenic corn versus soil insecticides led to rapid and widespread adoption of rootworm-Bt hybrids in the U.S. Corn Belt during the mid-2000s [2], which also significantly reduced insecticide applications and became the primary management tactic in continuous corn [27,31]. The pyramided Bt traits Cry3Bb1 + Cry34/35Ab1 [32], mCry3A + Cry34/35Ab1 [33,34], mCry3A + eCry3.1Ab [35], and Cry3Bb1 + Cry34/35Ab1 + DvSnf7 dsRNA [36] are the most recent rootworm-active trait registrations in the United States.

Greater than expected injury (i.e. root injury exceeding one node) as defined by the USEPA [27,37] to Cry3Bb1-expressing Bt hybrids has been documented in different areas of the Corn Belt since 2009 [31,38,39]. Miehls et al. [40] determined that under constant selection pressure in the laboratory, WCR evolved incomplete resistance to Cry3Bb1 in as few as three generations. A similar scenario was later observed in the field as WCR resistance evolved in fields planted to Cry3Bb1 for three or more consecutive years [31,38,41]. Cross-resistance between Cry3Bb1 and mCry3A or eCry3.1Ab has also been well documented [31,38,42]. Incomplete field-evolved resistance of WCR to Cry34/35Ab1 was first observed in 2013 from a few Iowa locations [43] and later from a Minnesota population [44].

Preserving the durability and efficacy of rootworm-active Bt proteins is of primary concern in agroecosystems because of the adaptability of WCR to different management tactics. Various laboratory bioassay techniques have been used to formally characterize WCR susceptibility to Bt traits [38,42,45]. To date, WCR bioassays have been used primarily to evaluate the susceptibility of populations from fields exhibiting greater than expected injury to confirm field-evolved resistance in various locations [31,38,41,42]. However, no studies have addressed the general spatial variation in WCR susceptibility to rootworm-Bt corn events at the local level (i.e. field to field variation within a county).

Within a landscape, many factors interact in an area over time to determine WCR susceptibility to rootworm-Bt traits. Examples include: length of time single-trait or pyramided trait hybrids are planted, rootworm densities in an area, field-evolved resistance to rootworm-Bt hybrids, potential gene flow of resistant or susceptible alleles, and frequency that other WCR tactics are used (e.g. crop rotation). Repeated use of single-trait Cry3Bb1 hybrids in Nebraska has selected for field-evolved resistance and associated cross-resistance to mCry3A in some populations [31]. In affected areas, various layers of tactics have been applied to mitigate or slow the spread of resistance. Greatly lowering WCR densities with alternative tactics such as crop rotation, insecticides, or efficacious traits protects yield, but may mask field-evolved resistance to Cry3Bb1 or mCry3A that is still present. For example, if a planting-time soil insecticide is applied to complement a failing single Cry3 trait (level of WCR resistance to trait is present), the insecticide will reduce larval injury to the root system [46,47] and often increase yield. However, selection pressure from the trait will continue as adults are produced from roots that grow outside of the insecticide-treated zone around the base of the plant. This will maintain or potentially increase the frequency of individuals that are resistant to the trait. Collectively, these factors make it increasingly difficult to track WCR trait susceptibility levels or the spread of rootworm resistance in an area over time. Therefore, understanding WCR susceptibility to Bt corn events at various spatial scales is important to preserve susceptibility to current technologies and increase understanding of Bt resistance within the landscape. To address this, adult sampling grids were established in Keith and Buffalo counties, Nebraska, and single-plant larval bioassays were conducted with progeny from adult field collections to characterize susceptibility to Cry3Bb1 and mCry3A proteins. A retrospective approach was used to evaluate the relationship of past rootworm management practices to the current susceptibility of rootworm collections. Specific objectives included: 1) characterize spatial variability in susceptibility of WCR field populations to Cry3Bb1 and mCry3A in two corn production areas of Nebraska; 2) indirectly document gene flow of resistant alleles within the grid systems; and 3) characterize the relationship between past WCR management practices and current WCR susceptibility levels. These real-world case histories will inform the use of current rootworm-Bt products and future technologies that will be deployed.

## Materials and methods

### Sampling grid system

**Fig 1:**
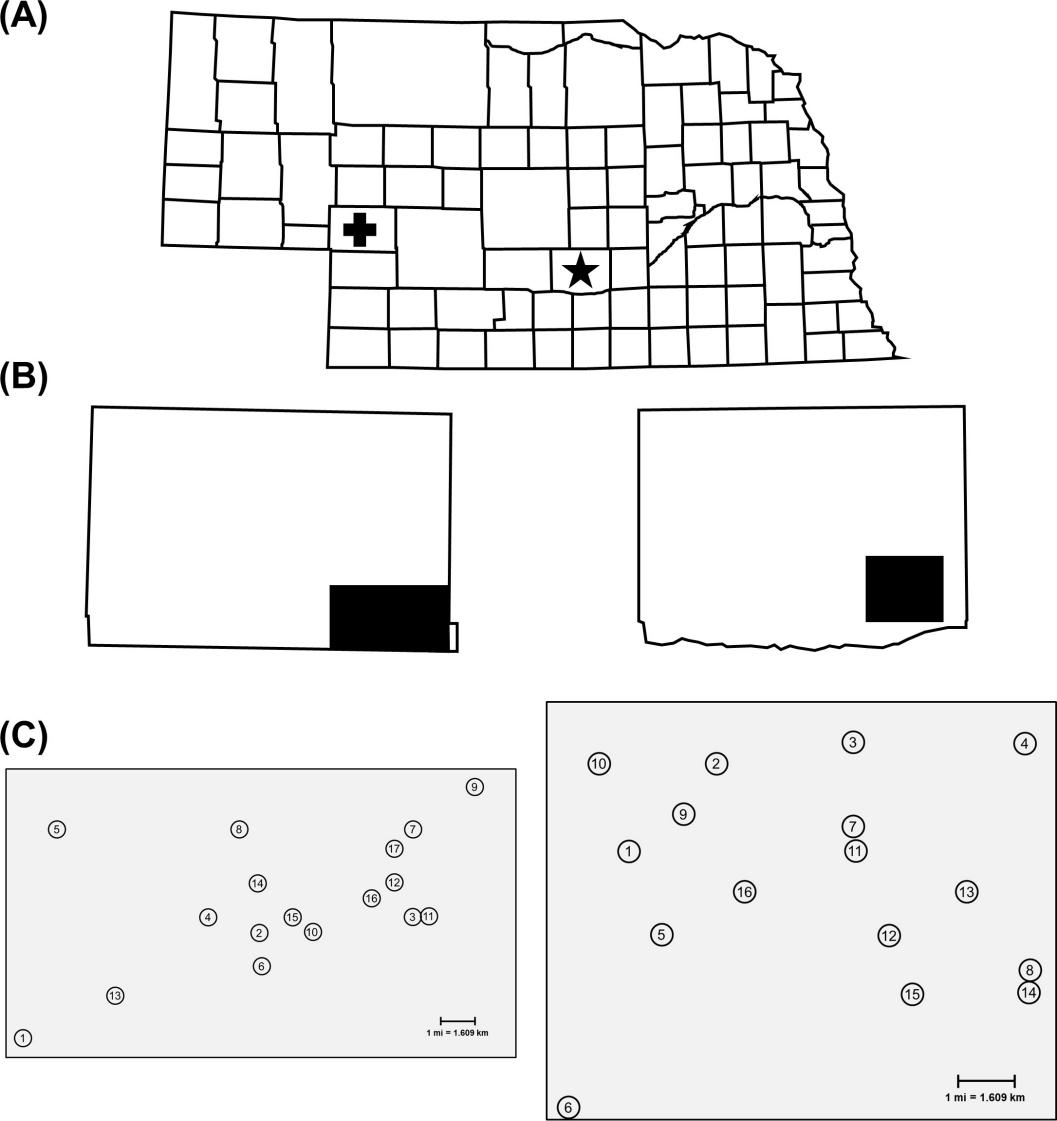
Sampling grid system established in two Nebraska counties. (A) County map of the state of Nebraska showing location of Keith County (+) and Buffalo County (*). (B) Areas of Keith County (left) and Buffalo County (right) where sampling grids were established. (C) Spatial distribution of sampling sites in Keith County (left) and Buffalo County (right).

In 2015, initial sampling grids were established in Keith and Buffalo counties, Nebraska (Fig 1A–1C). Within grids, each field was assigned a unique number that is consistently used in all tables and figures in this paper (Fig 1C). These counties are two high-yielding, intensive corn production areas of the state. A high proportion of fields within both grids were planted to continuous corn under center pivot irrigation, often producing moderate to high rootworm population densities. Individual fields 3–8 km apart were systematically selected in 2015 to characterize susceptibility across a defined area of each county. Most fields had been planted to corn for at least two years, but some fields were selected because Cry3Bb1- or mCry3A-expressing Bt hybrids had not been used. In 2016, additional field sites were collected in each defined sampling grid to increase the total sample size per grid and to characterize susceptibility on a smaller scale within each grid. Access to fields to make WCR collections was allowed by owners (private or University). This study did not involve any endangered or protected species.

The Keith County grid, located south of Paxton, NE, contained 17 total field sites in a 10 by 20 km area with sampling sites 2–8 km apart (Fig 1B and 1C). The majority of the Keith County grid was characterized by continuous corn planted to Bt-expressing hybrids as single-trait or pyramided products. Cry3Bb1- and mCry3A-expressing hybrids were more commonly planted for multiple years in the northern than the southern half of the grid, and the southern half included five fields in which neither trait had been planted (Table 1). Previous research confirmed the presence of field-evolved WCR resistance to Cry3Bb1 in some Keith County fields [31].

**Table 1:** Yearly trait history and cumulative index value of Keith County populations.

Year	Field			Trait History^a^					
Collected	Number	2010	2011	2012	2013	2014	2015	2016	Index^b^
**2015**	1^c^	0	0	0	0	0	0		0.6
	2^c^	0	0	0	0	0	0		1.6
	3^c^	0	0	0	0	0	0		0.1
	4	NA	2	2	5	2*	2*		1
	5	NA	NA	NA	4	2	2		2.1
	6	1	1	1	1	2*	1*		9
	7	5	4	3	1*	1	1		6.7
	8	4	0	2	1	2	1		9.2
	9	NA	NA	NA	2	2	2		6
**2016**	1^c^	0	0	0	0	0	0	0	0.7
	2^c^	0	0	0	0	0	0	3	1.7
	10^c^	0	0	0	0	0	0	3	1.2
	11^c^	0	0	0	0	0	0	0	0.2
	12	2	5	4	1*	1	1*	2*	6.6
	13	1	1	1	1	1*	1*	1*	11.0
	14	1	1	0	2	1*	1*	2	9.6
	15	1	1*	1	1*	2*	0	2	8.6
	16	2	1	1	1*	1	1*	3	10.6
	17	1	0	1	1	1*	1*	1*	9.6

^a^0 = no rootworm-Bt traits; 1 = single-trait Cry3Bb1; 2 = single-trait mCry3A; 3 = single-trait Cry34/35Ab1; 4 = popcorn; 5 = rotated to soybeans;

* = denoted trait was part of pyramided hybrid also containing Cry34/35Ab1; NA = field history not available for specified year.

^b^Criteria for total field history index values are available in Table 2.

^c^Field never planted to Cry3Bb1- or mCry3A-expressing hybrids

**Table 2:** Field history index values and criteria used to assign values to individual fields.

Category	Index Value^a^	Criteria
**Hybrid Values**^b^	0.1	Popcorn, conventional, or single-trait Cry34/35Ab1 hybrid planted
	0.5	Pyramided Cry3Bb1 or mCry3A hybrid planted
	1.0	Single-trait Cry3Bb1 or mCry3A hybrid planted
**Selection Values**^c^	3.0	3 years of single-trait/pyramided Cry3Bb1 or mCry3A hybrid planted
	0.5	Pyramided Cry3Bb1 or mCry3A hybrid planted after initial 3 years
	1.0	Single-trait Cry3Bb1 or mCry3A hybrid planted after initial 3 years
**Crop Rotation**	RESET	All previous history values excluded when crop rotation occurred.
		Values were assigned the year(s) following rotation.
**Area Effect**	0.5	Multiple resistant populations, one susceptible population within 1 mile
	1.0	Resistant population(s) within 1 mile
	-0.5	Multiple susceptible populations, one resistant population within 1 mile
	-1.0	Susceptible population(s) within 1 mile

^a^All values per site were additive to obtain an overall index value used in statistical analyses

^b^Hybrid values were assigned per growing season based on use in individual fields

^c^The initial selection value of 3.0 was assigned after 3 consecutive years of Cry3 trait use. Additional selection values of 0.5 and 1.0 were assigned per growing season based on use of Cry3 traits after the initial 3 years of selection.

The Buffalo County grid, located between Gibbon and Kearney, NE, consisted of 16 total field sites in an 11 by 13 km area; sampling sites were 2–5 km apart (Fig 1B and 1C). Field histories indicated that use of multiple integrated pest management (IPM) tactics and non-Bt hybrids was more common in the western portion of the grid (Table 3). In contrast, the eastern portion of the grid was characterized by more Cry3Bb1 or mCry3A use, and greater than expected injury was reported in the northeast portion of the grid in 2012–2013. Similar to the Keith County grid, four fields were included where Cry3Bb1- or mCry3A-expressing hybrids had never been planted. In both counties, fields never planted to Cry3Bb1- or mCry3A-expressing hybrids served as field control sites and larval bioassay data from these fields were used to determine the potential for gene flow to spread resistant alleles within the landscape.

**Table 3:** Yearly trait history and cumulative index value of Buffalo County populations.

Year	Field			Trait History^a^					
Collected	Number	2010	2011	2012	2013	2014	2015	2016	Index^b^
**2015**	1^c^	0	0	0	0	0	0		0.6
	2^c^	0	0	3	5	0	0		0.2
	3^c^	NA	5	0	0	3	0		0.4
	4	1	5	0	1	5	0		1.1
	5^c^	0	0	5	0	0	0		0.3
	6	0	5	0	0	2	2		2.2
	7	NA	NA	NA	NA	NA	NA		X
	8	NA	NA	NA	NA	NA	NA		X
**2016**	9	0	0	0	0	0	0	2	1.6
	10	NA	NA	0	1	5	0	2	1.1
	11	0	0	0	5	0	2	2	2.1
	12	0	0	1	5	2	0	1*	2.6
	13	NA	NA	1	5	0	2	2	2.1
	14	0	0	0	0	0	1*	1*	1.5
	15	1	1	0	2	2	1*,2*	2	9.6
	16	1	1	0	0	0	0	2	3.4

^a^0 = no rootworm-Bt traits; 1 = single-trait Cry3Bb1; 2 = single-trait mCry3A; 3 = single-trait Cry34/35Ab1; 4 = popcorn; 5 = rotated to soybeans;

* = denoted trait was part of pyramided hybrid also containing Cry34/35Ab1; NA = field history not available for specified year.

^b^Criteria for total field history index values are available in Table 2.

^c^ Field never planted to Cry3Bb1- or mCry3A-expressing hybrids

### Field history index

Field histories dating back to 2010 were collected in 2015 and 2016 to evaluate the potential impact of past management practices on current WCR susceptibility levels to Cry3 toxins (Tables 1 and 3). The exceptions were field sites 7 and 8 in Buffalo County where field histories were unavailable. A field history index was created from weighted past WCR management variables and agronomic practices and assigned to individual fields in each grid (Table 2). The rating system was created to quantify the potential level of selection pressure placed on a population by the past management tactics deployed within individual fields. The working hypothesis was that as index values increase, WCR susceptibility to Cry3Bb1 or mCry3A will decrease. Values used to create the field history index were additive and based on current knowledge of the agronomic system in Nebraska and the potential effects of management practices on WCR susceptibility levels to Cry3 toxins gleaned from published literature.

The first step when assigning an index value to a specific field was to determine the Bt traits planted from 2010 to collection year and assign hybrid values per year (Table 2). A value of 1.0 was assigned for single-trait Cry3-expressing hybrids and a value of 0.5 was assigned for pyramided hybrids containing Cry3 toxins per year. These values were weighted because evolution of resistance often occurs faster with single-trait versus pyramided hybrids [48]. Cry3Bb1 and mCry3A were treated equally because of cross-resistance between them [31,41,42]. Popcorn, conventional hybrids, and single-trait Cry34/35Ab1 were assigned a value of 0.1. Although selection pressure for Cry3Bb1 or mCry3A toxins is absent when non-rootworm-Bt hybrids or Cry34/35Ab1-expressing hybrids are planted, resistant alleles present from prior selection should be maintained in the WCR population emerging from the field [40,49] because there is little evidence of a fitness cost of resistance to Cry3 traits [50,51]. Popcorn is usually planted after rotation from a non-WCR host in Nebraska, so no selection pressure would be applied and few WCR would be produced. In addition to yearly hybrid values, fields planted to Cry3-expressing hybrids for 3 consecutive years were assigned 3 additional points to account for a potential susceptibility shift. This was based on multiple field reports of field-evolved resistance to Cry3Bb1 after planting Cry3Bb1-expressing hybrids for 3 or more consecutive years [31,38]. Additional selection values of 1.0 and 0.5 were assigned per growing season based on use of single-trait or pyramided Cry3 hybrids, respectively, after the initial 3 years of selection (Table 2).

The rotation of field corn with a non-host plant, such as soybeans (*Glycine max* L.) or pinto beans (*Phaseolus vulgaris* L.), is an effective WCR management tactic in the western Corn Belt because oviposition takes place primarily in corn and eggs overwinter in the soil until the following growing season [15,17]. When crop rotation occurred after 2010, all index values previously assigned were excluded from the total index summary; values were only assigned to years following rotation (Table 2). Crop rotation “resets” the field—first-year cornfields often have undetectable to very low WCR populations, eliminating most resistant alleles within the field [52–54].

The impact of potential beetle movement from surrounding fields was also taken into account in the field history index (Table 2). The area effect category of the field history index was based on incomplete knowledge of all fields, but recolonization of first-year cornfields routinely occurs [55]. The potential of WCR exhibiting trivial or migratory flight [15,56,57] may facilitate gene flow among fields [15]. Fields within one mile of a potential resistant population were assigned a value of 1.0, while fields within one mile of a potential susceptible population were deducted a value of 1.0. If multiple resistant populations and one known susceptible population occurred within one mile, a value of 0.5 was added; if multiple susceptible populations and one resistant population occurred within one mile, a value of 0.5 was deducted. For example, Keith County field site 12 (Table 1) was planted to single-trait mCry3A in 2010 (value of 1.0; total = 1.0). This field was rotated to soybeans the following year, resetting values from past years (total = 0.0). Popcorn was planted in 2012 following the soybean rotation (value of 0.1; total = 0.1). A pyramided Cry3Bb1 hybrid was planted in 2013 (value of 0.5; total = 0.6), a single-trait Cry3Bb1 hybrid was planted in 2014 (value of 1.0; total = 1.6), and a pyramided Cry3Bb1 hybrid was planted in 2015 (value of 0.5; total = 2.1). Because three consecutive years of selection with Cry3Bb1-containing hybrids occurred, the selection value of 3.0 was added (total = 5.1). A pyramided hybrid containing mCry3A was planted in 2016 (value of 0.5; total = 5.6), adding an additional year of Cry3 selection (value of 0.5; total = 6.1). Because this field site was within one mile of multiple resistant field sites and one susceptible field site, an area effect value of 0.5 was assigned (total field history index for field site 12 = 6.6). Field history index derivations for each field can be found in the supplemental material (S1 Table).

### Adult collections

Beetles were initially collected from 8 different fields in Buffalo County (collected 6 August 2015) and 9 different fields in Keith County (collected 24–25 August 2015). Eight additional fields were sampled in both Buffalo County (collected 2 September 2016) and Keith County (collected 3–4 August 2016) the following year (Fig 1C). In Keith County, different areas within fields 1 and 2 were also sampled each year. A minimum of 50 gravid females (range: 50–250) were collected per field to obtain an adequate subset of the natural variation present. In conjunction with beetle collection, lateral flow strips (Envirologix Inc., Portland, ME) were used to confirm the expression of one or more Bt traits in each field. Adults were brought to the laboratory at the University of Nebraska-Lincoln and maintained by field in 28cm^3^ plexiglass cages to obtain eggs. The standard rearing procedure and temperature profile for holding eggs to facilitate diapause development and termination are described in Wangila et al. [31].

Four diapausing WCR colonies collected before commercialization of Bt corn hybrids in 2003 were used as controls in 2016 and 2017 laboratory bioassays (no prior exposure to Bt corn). Populations were collected from Butler County, Nebraska (1990), Potter County, South Dakota (1995), Finney County, Kansas (2000), and Centre County, Pennsylvania (2000). These colonies were maintained at the USDA North Central Agricultural Research Laboratory in Brookings, South Dakota.

A WCR field population collected during 2015 and 2016 from the same field at the Eastern Nebraska Research and Extension Center, Ithaca, Nebraska, was also used as a field control in bioassays. Large areas of continuous corn without rootworm-Bt traits were annually planted around the field so a large refuge was maintained. Hybrids expressing rootworm-active traits were only periodically planted in small-plot research trials at this site so WCR exposure to rootworm traits was minimal.

### Single-plant larval bioassays

Single-plant larval bioassays following the procedure described by Gassmann et al. [38] and adapted by Wangila et al. [31] were used to document variation in susceptibility to Cry3Bb1 and mCry3A in both sampling grids. Data from the bioassays were used in different ways to address the three objectives of this study. Bioassays were conducted at the University of Nebraska-Lincoln in spring and summer of 2016 and 2017. Control populations were assayed at various time points throughout the period bioassays were conducted. During both years, Cry3Bb1 (Stone 6021VT3) and mCry3A (Syngenta N68B-3000GT) expressing hybrids and their non-transgenic near-isolines (Stone 6021RR2 and Syngenta N68B-GT, respectively), hereafter termed isoline, were used in bioassays. A neonicotinoid seed treatment applied to all seeds at 0.25 mg ai/kernel was removed prior to bioassays using the procedure described in Gassmann et al. [38].

Corn plants were grown under greenhouse conditions in 1-L clear plastic containers (Johnson Paper & Supply Co., Minneapolis, MN), filled with a 1:1 mixture of Sun Gro Metro-Mix 900 Grower Mix and Sun Gro Sunshine LC1 Grower Mix (Sun Gro Horticulture, Inc., Agawam, MA) following the methods outlined in Wangila et al. [31]. One seed was planted per pot, and 12 pots of each hybrid were organized in a randomized complete block design. Supplemental lighting (400W 208volt Jasond light bulbs, P. L. Light Systems, Beamsville, ON, Canada) beyond the natural photoperiod was provided as needed to reach a 14:10 (L:D) h cycle. Average daily low and high temperatures ranged from 19.9–33.2°C and 18.7–30.8°C in 2016 and 2017, respectively. MiracleGro Water Soluble All Purpose Plant Food (The Scott’s Company LLC, Pacific Junction, IA) was applied at the V2 growth stage [58] at a ratio of 4mg: 1mL of water; 100mL was applied to each plant.

Bioassays were conducted with V4 to V5 growth stage plants [58]. Each plant was infested with 12 neonate larvae less than 24h after eclosion. A small hole was dug in the soil media to expose root tissue, and each larva was individually placed on roots with a size 20/0 soft hair brush to facilitate maximum initial larval colonization. Roots were re-covered with soil media after infestation. Plant foliage was cut 20cm above the soil prior to placement in growth chambers (Percival Scientific Inc., Perry, IA) at 24°C with a 14:10 (L:D) h photoperiod. To facilitate larval establishment, water was not applied until 3 days post-infestation and then 50mL was applied every other day to maintain corn vigor. Plants were organized in a randomized complete block design in growth chambers to account for potential variability in temperature and lighting. Preliminary bioassays and results from Wangila et al. [31] indicated that under these growth chamber conditions, terminating bioassays on day 17 would ensure larvae did not reach the pre-pupal stage. After 17 days, the remaining above-ground plant mass was cut off at the soil line before the soil/root mass was placed into a Berlese funnel (40W, 120V bulbs–Philips Lighting Company, Worcester, MA) for 4 days. Larval survivors were collected in 70% ethyl alcohol and subsequently counted.

### Data analysis

A generalized linear model assuming a beta distribution [59,60] and a two-way treatment structure implemented in PROC GLIMMIX [61] was used to compare mean proportional survival from bioassays between each Bt and its associated isoline hybrid. Fixed factors included population (field site), corn hybrid (Bt or respective isoline), and the interaction between population and corn hybrid. A mean lab control value from the four lab control populations was used in the analyses as preliminary analyses indicated proportional survival on Cry3Bb1- or mCry3A-expressing hybrids was not significantly different among lab control populations within bioassay years (data in S2 Table). Paired comparisons between hybrids within populations were conducted using the LSMEANS SLICEDIFF option to identify significant differences in bioassay survivorship (*P* = 0.05). Tukey’s adjustment for multiple comparisons was used to control type I error.

To obtain a composite picture of geographic variation in WCR susceptibility to each trait at several spatial scales, survivorship data were pooled within traits over years. Pooling data over years was supported by a number of factors. Rearing and bioassays were conducted under similar conditions and time of year, plus the same lab and field control populations were used both years. Also, survivorship on lab and field controls, respectively, was not significantly different between years (data in S3 Table).

To evaluate small-scale field to field variation in WCR susceptibility to Cry3Bb1 and mCry3A and create geographical maps documenting this variation, pooled 2016–2017 survivorship data excluding lab and field controls were analyzed by trait and county using a generalized linear model assuming a beta distribution and implemented with PROC GLIMMIX [61]. Population was included in the model as a fixed factor. The LSMEANS PDIFF option was used to identify significant differences in survival among populations. Tukey’s adjustment for multiple comparison was used to control type I error.

Larger spatial scale comparison of pooled 2016–2017 WCR proportional survival by trait across groups of fields within each grid were also evaluated. Fields in the Keith County grid were divided into north versus south areas and the Buffalo County grid into east versus west areas based on trends obtained from past trait use histories compiled for each grid. A generalized linear model assuming a beta distribution (PROC GLIMMIX [61]) was used to determine geographic differences in susceptibility between categories in each grid. Geographic location was included in the model as a fixed factor. The LSMEANS statement was used to identify the mean and standard error of each area of the sampling grid, and the PDIFF option was used to determine significant differences between locations.

A scoring analysis was used to estimate survivorship values from 2017 bioassays based on survivorship and field history index data from 2016 bioassays [62]. PROC GLIMMIX [61] was used to fit a generalized linear model with an ANCOVA structure to test for equal slopes to determine if data from both counties and bioassay years could be pooled. County and bioassay year were included in separate analyses as a covariate to determine if either variable impacted the relationship between field history index and proportional survival. The county*field history index interaction (F = 0.05; df = 1, 29 *P =* 0.8227) and the year*field history index interaction (F = 3.36; df = 1, 29 *P =* 0.0773) were not significant, indicating equal slopes and that the relationship between field history index values and proportional survival did not differ based on county or bioassay year. Therefore, pooled Keith and Buffalo county bioassay data were categorized by year to enable use of 2016 data to estimate 2017 data. A dataset containing only 2016 values was created and PROC GLIMMIX [61] was used to fit a beta generalized linear regression model. The STORE statement was used to save the context and results of the analysis. PROC PLM was used to score the 2017 dataset using the previous model fit to the 2016 dataset. Predicted survivorship values were calculated based on the model produced by the 2016 dataset and back-transformed to the data scale using the ILINK option. Actual versus predicted survivorship was plotted using PROC GPLOT and PROC CORR (Pearson correlation coefficient) was used to determine the strength of relationship between the predicted and actual survivorship values.

Sensitivity analyses were conducted by manipulating field history index category values or removing entire categories from the base model to understand the importance of each variable in predicting current WCR susceptibility levels (data in S4 Table). The data were re-analyzed using the scoring analysis outlined above after adjusting field history index values for each field. Because the scoring analysis produces a different regression equation for each adjusted field history index, the correlation r values between actual and predicted Cry3Bb1 survivorship from the adjusted field history index were compared to the correlation value from the original field history index (Table 2) by testing the difference between two unpaired correlations using a paired.r test [63] (data in S4 Table).

## Results

### Survivorship on transgenic and non-transgenic hybrids

#### Keith County grid

**Fig 2:**
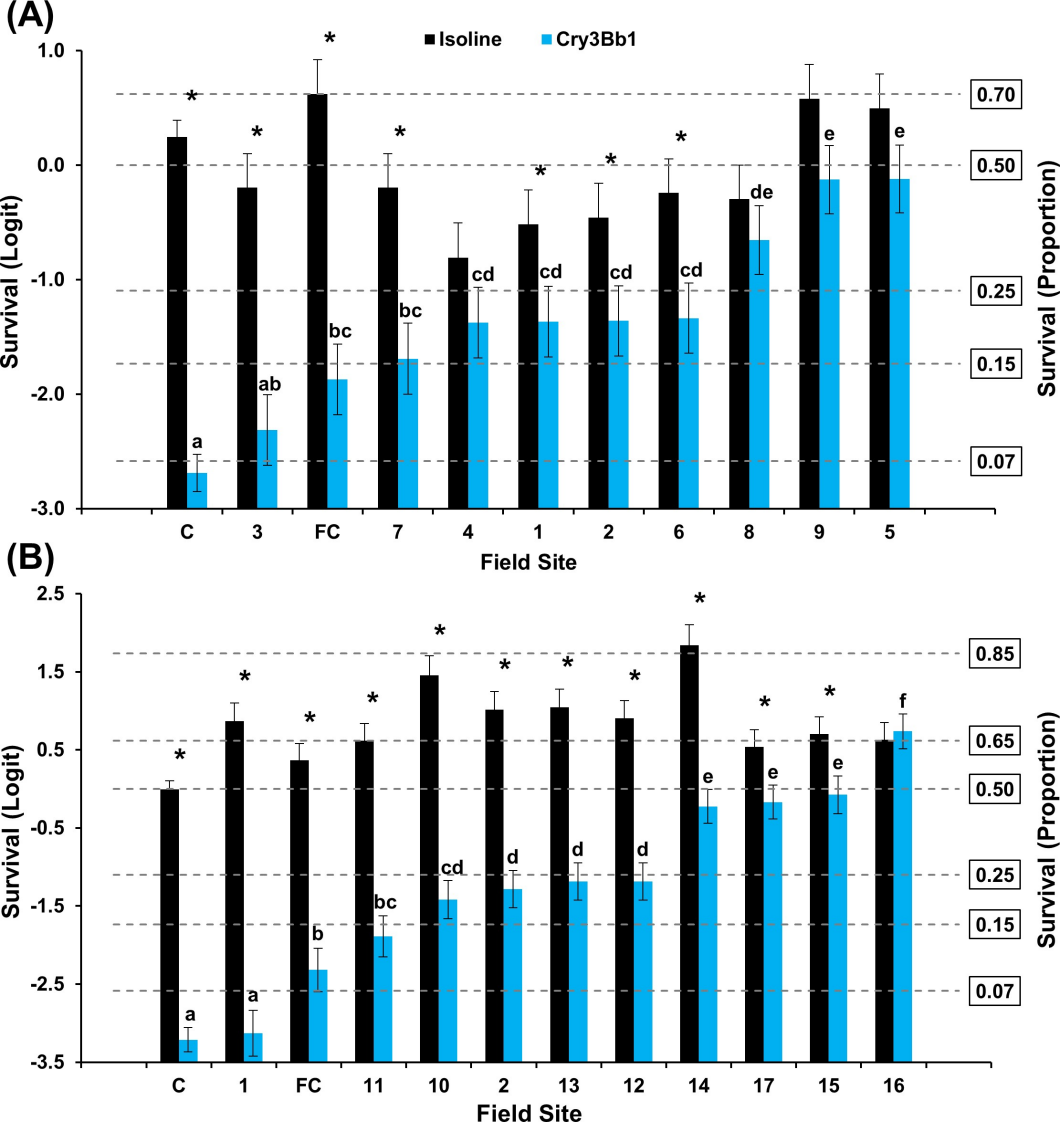
Mean WCR larval survival (± SE) of Keith County populations on Cry3Bb1 and its isoline. (A) 2016 bioassays. (B) 2017 bioassays. C = composite lab control; FC = field control. * indicates significantly higher survival on isoline compared to Cry3Bb1. Within years, Cry3Bb1 means with the same letter were not significantly different (generalized linear model, *P* > 0.05; LSMEANS option).

A significant interaction between population and hybrid occurred for populations assayed on Cry3Bb1 and its non-Bt near-isoline in 2016 (F = 7.40; df = 10, 314; *P*<0.0001) and 2017 (F = 13.92; df = 11, 336; *P*<0.0001) (Fig 2). A significant difference in mean survivorship on Cry3Bb1 was documented among populations in both years. Mean survivorship on Cry3Bb1 ranged from 9–47% and 4–68% in 2016 and 2017, respectively. Mean survival of one population was not significantly different than the mean lab control each year. In 2016 bioassays, mean survival of six populations was not significantly different than mean survival of the field control, while only one population exhibited this characteristic in 2017 bioassays (Fig 2). Within populations, mean survival on the isoline was significantly greater than survival on Cry3Bb1 in five populations in 2016 and nine populations in 2017 (Fig 2). Mean survival between hybrids was not significantly different within the remaining 2016 and 2017 populations.

**Fig 3:**
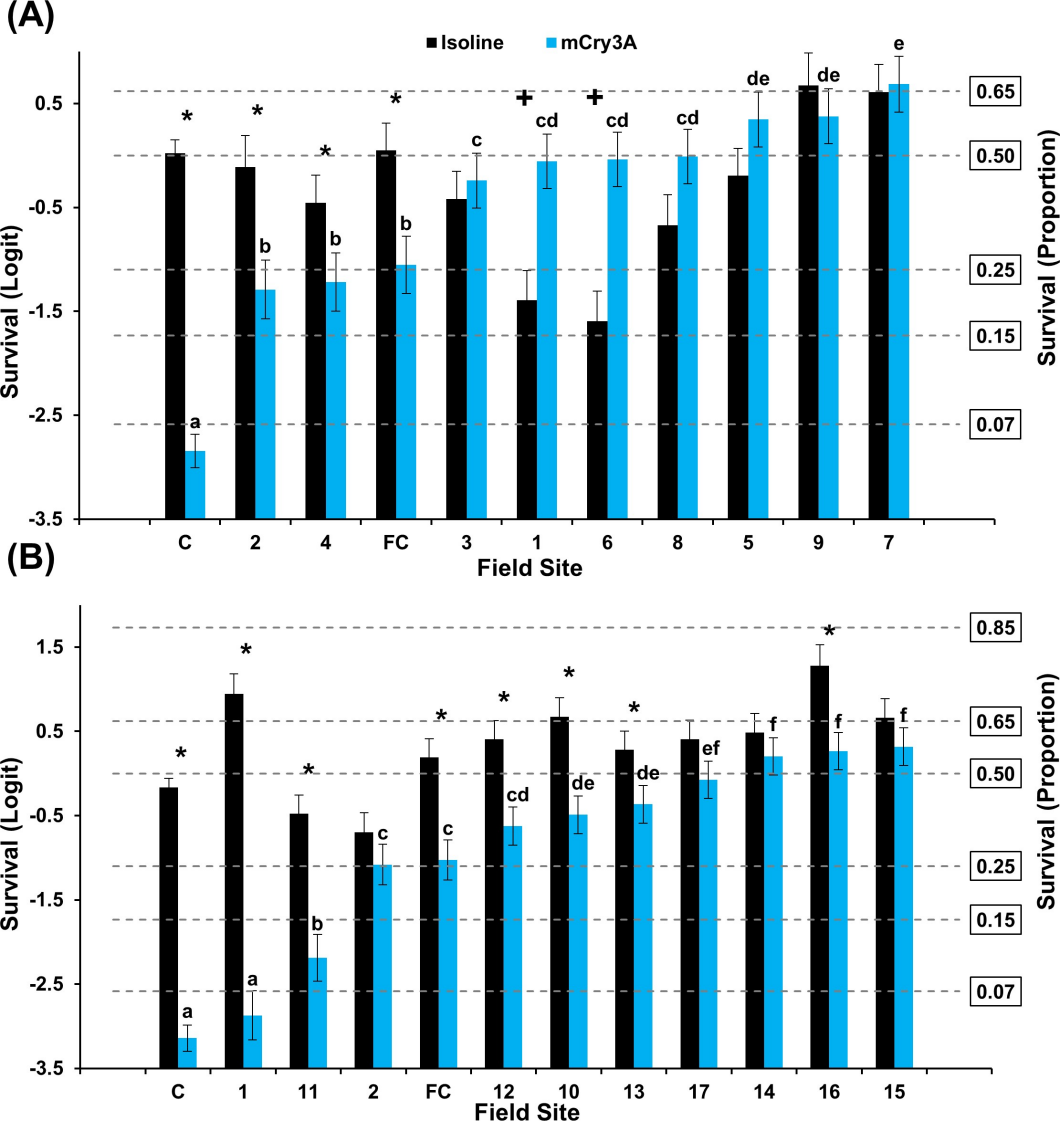
Mean WCR larval survival (± SE) of Keith County populations on mCry3A and its isoline. (A) 2016 bioassays. (B) 2017 bioassays. C = composite lab control; FC = field control. * indicates significantly higher survival on isoline compared to mCry3A. + indicates significantly higher survival on mCry3A compared to the isoline. Within years, mCry3A means with the same letter were not significantly different (generalized linear model, *P* > 0.05; LSMEANS option).

The results of mCry3A bioassays followed a pattern similar to that observed in Cry3Bb1 bioassays. A significant interaction occurred between population and hybrid for populations assayed on mCry3A and its isoline in 2016 (F = 19.82; df = 10, 306; *P*<0.0001) and 2017 (F = 15.55; df = 11, 336; *P*<0.0001) (Fig 3). Significant differences in mean survivorship occurred among populations, with survival levels ranging from 22–67% in 2016 and 6–58% in 2017 (Fig 3). In 2016, all populations exhibited significantly greater survival on mCry3A compared to the mean lab control (Fig 3). With one exception, a similar trend was observed in 2017. Mean survival of two populations each in 2016 and 2017 bioassays were not significantly different than the field control (Fig 3). Within Keith County populations, mean survival on the isoline was significantly greater than survival on mCry3A in two populations during 2016 bioassays and six populations in 2017 (Fig 3). Two populations also exhibited significantly greater mean survival on mCry3A compared to the isoline in 2016 (Fig 3).

#### Buffalo County grid

**Fig 4:**
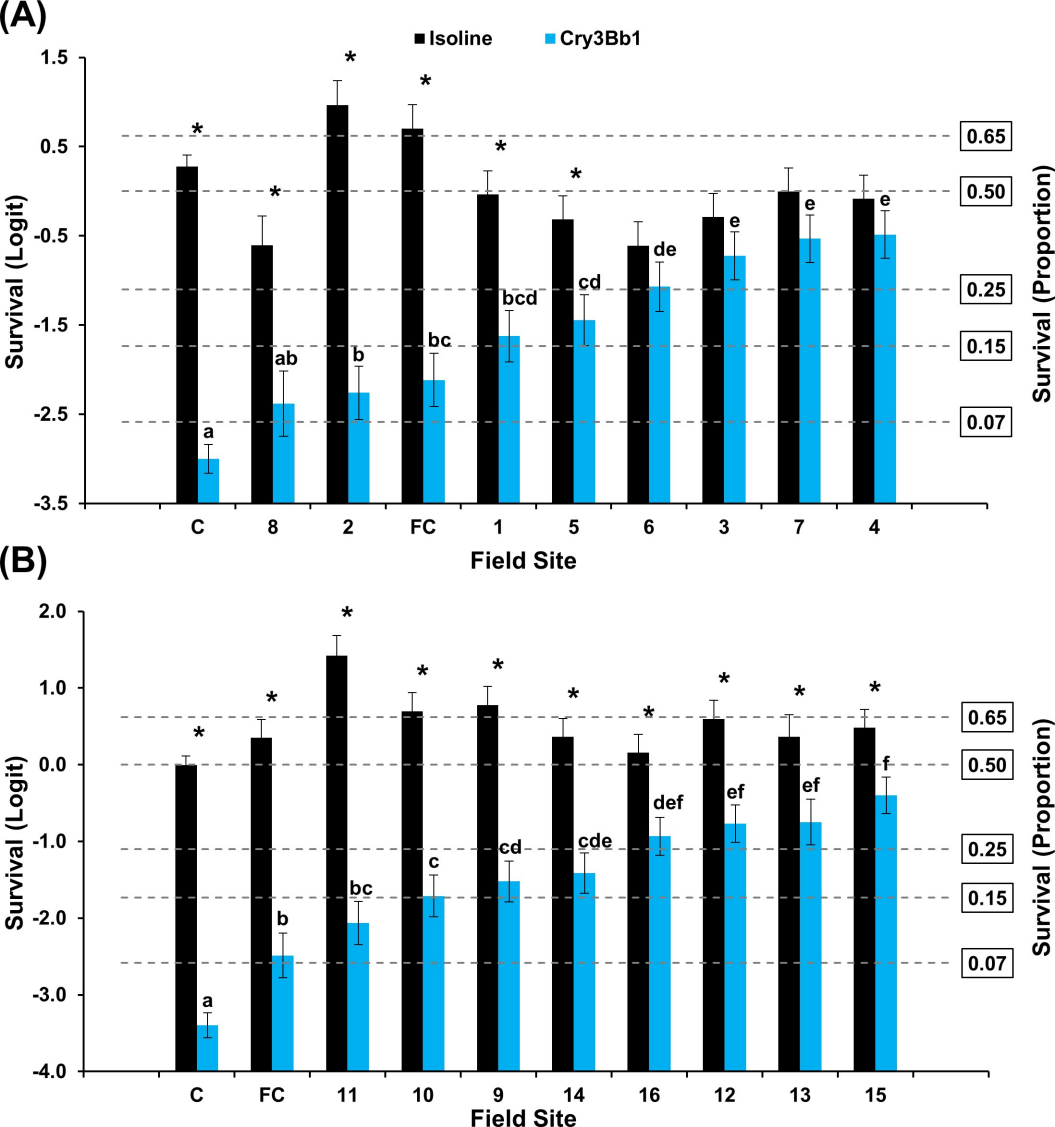
Mean WCR larval survival (± SE) of Buffalo County populations on Cry3Bb1 and its isoline. (A) 2016 bioassays. (B) 2017 bioassays. C = composite laboratory control; FC = field control. * indicates significantly higher survival on isoline compared to Cry3Bb1. Within years, Cry3Bb1 means with the same letter were not significantly different (generalized linear model, *P* > 0.05; LSMEANS option).

A significant interaction between population and hybrid was documented in populations assayed with Cry3Bb1 and isoline corn in 2016 (F = 13.32; df = 9, 284; *P*<0.0001) and 2017 (F = 10.00; df = 9, 284; *P*<0.0001) (Fig 4). The general pattern detected among populations in Buffalo County was consistent with the results from Keith County. Mean survivorship ranged from 8–38% in 2016 and 11–40% in 2017. All but one population assayed during 2016–2017 exhibited significantly higher survival on Cry3Bb1 than the lab control (Fig 4). Mean survival on Cry3Bb1 of four populations bioassayed in 2016 and one population bioassayed in 2017 was not significantly different than the mean field control (Fig 4). Within populations, mean survival on the isoline was significantly greater than survival on Cry3Bb1 in four populations assayed in 2016 and all populations assayed in 2017 (Fig 4).

**Fig 5:**
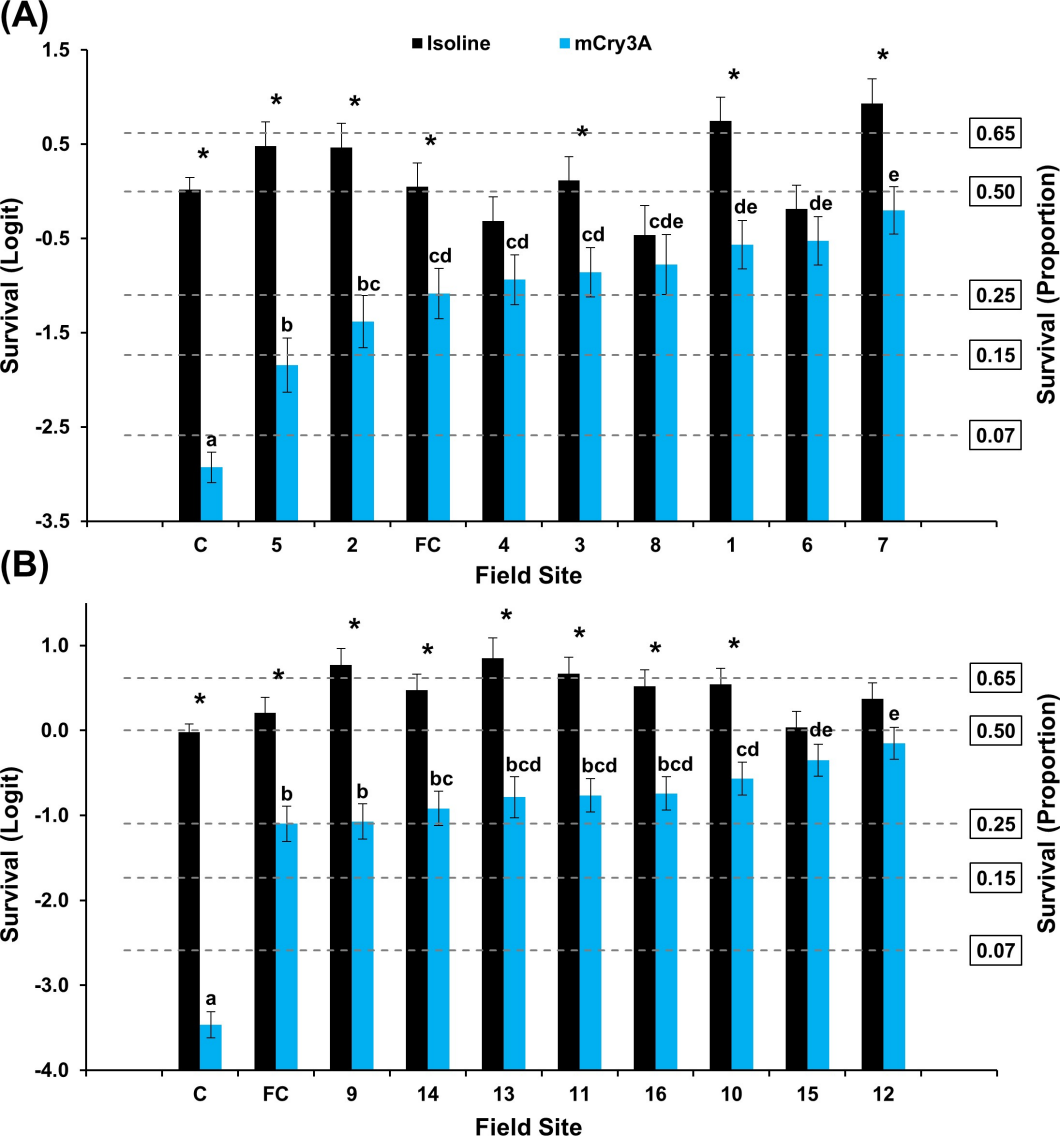
Mean WCR larval survival (± SE) of Buffalo County populations on mCry3A and its isoline. ((A) 2016 bioassays. (B) 2017 bioassays. C = composite laboratory control; FC = field control. * indicates significantly higher survival on isoline compared to mCry3A. Within years, mCry3A means with the same letter were not significantly different (generalized linear model, *P* > 0.05; LSMEANS option).

A significant interaction between population and hybrid occurred for populations assayed on mCry3A and its isoline in 2016 (F = 9.69; df = 9, 284; *P*<0.0001) and 2017 (F = 16.57; df = 9, 284; *P*<0.0001) (Fig 5). Mean survivorship ranged from 14–45% in 2016 and 26–46% in 2017. All populations exhibited significantly greater survival on mCry3A compared to the lab control in 2016 and 2017 assays. Mean proportional survival of six field populations bioassayed in 2016 and five populations bioassayed in 2017 was not significantly different than the field control (Fig 5). Within populations, mean survival was significantly greater on isoline than mCry3A in five and six populations in 2016 and 2017, respectively (Fig 5).

### Geographic variation in Cry3 susceptibility

#### Keith County grid

**Fig 6:**
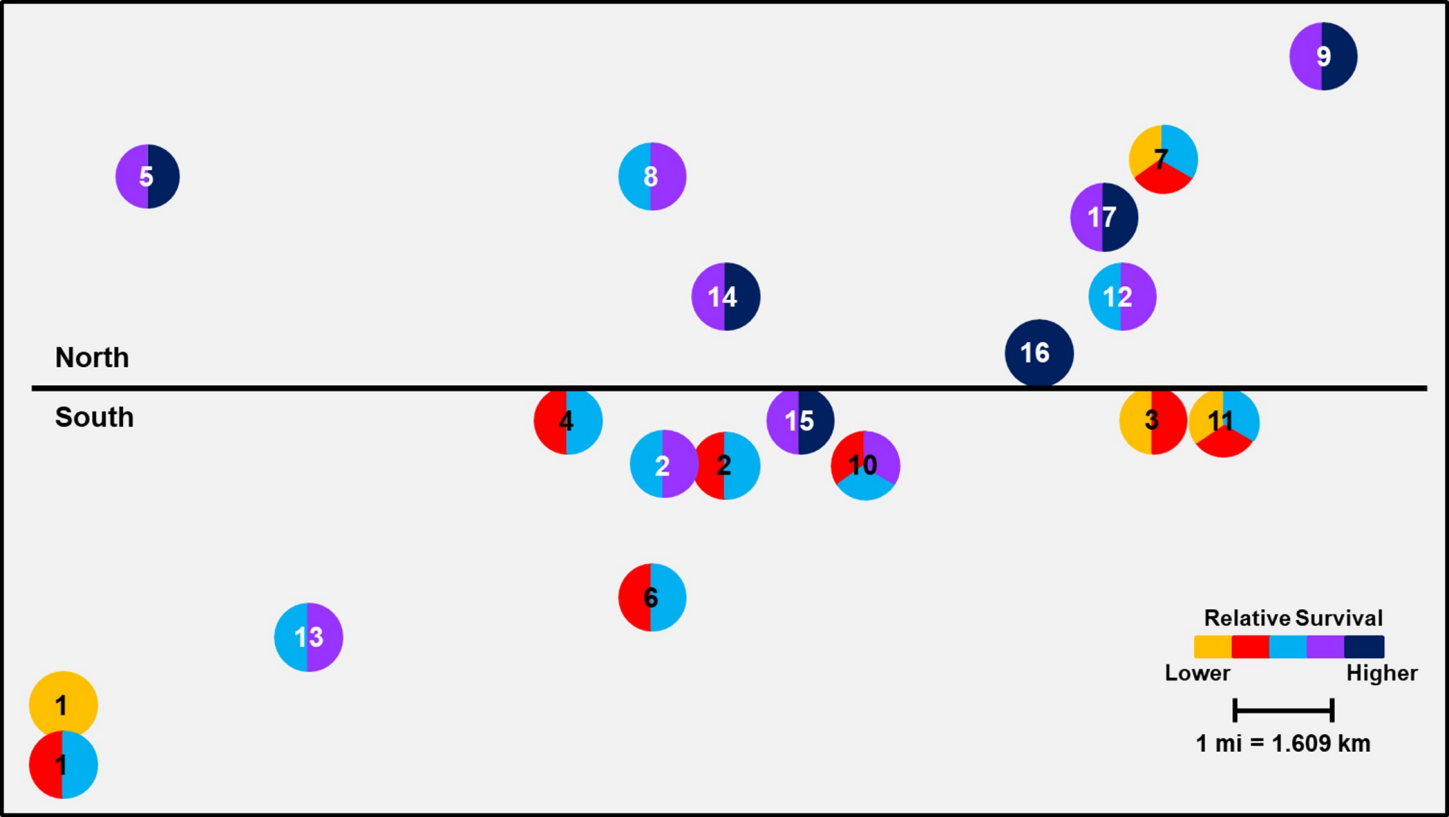
Spatial variation in mean Cry3Bb1 survival of WCR larvae among sampling sites in Keith County. Mean proportional survival ranged from 0.04 ± 0.01 to 0.66 ± 0.06. Circles containing the same color(s) are not significantly different (generalized linear model, P > 0.05; LSMEANS option).

**Fig 7:**
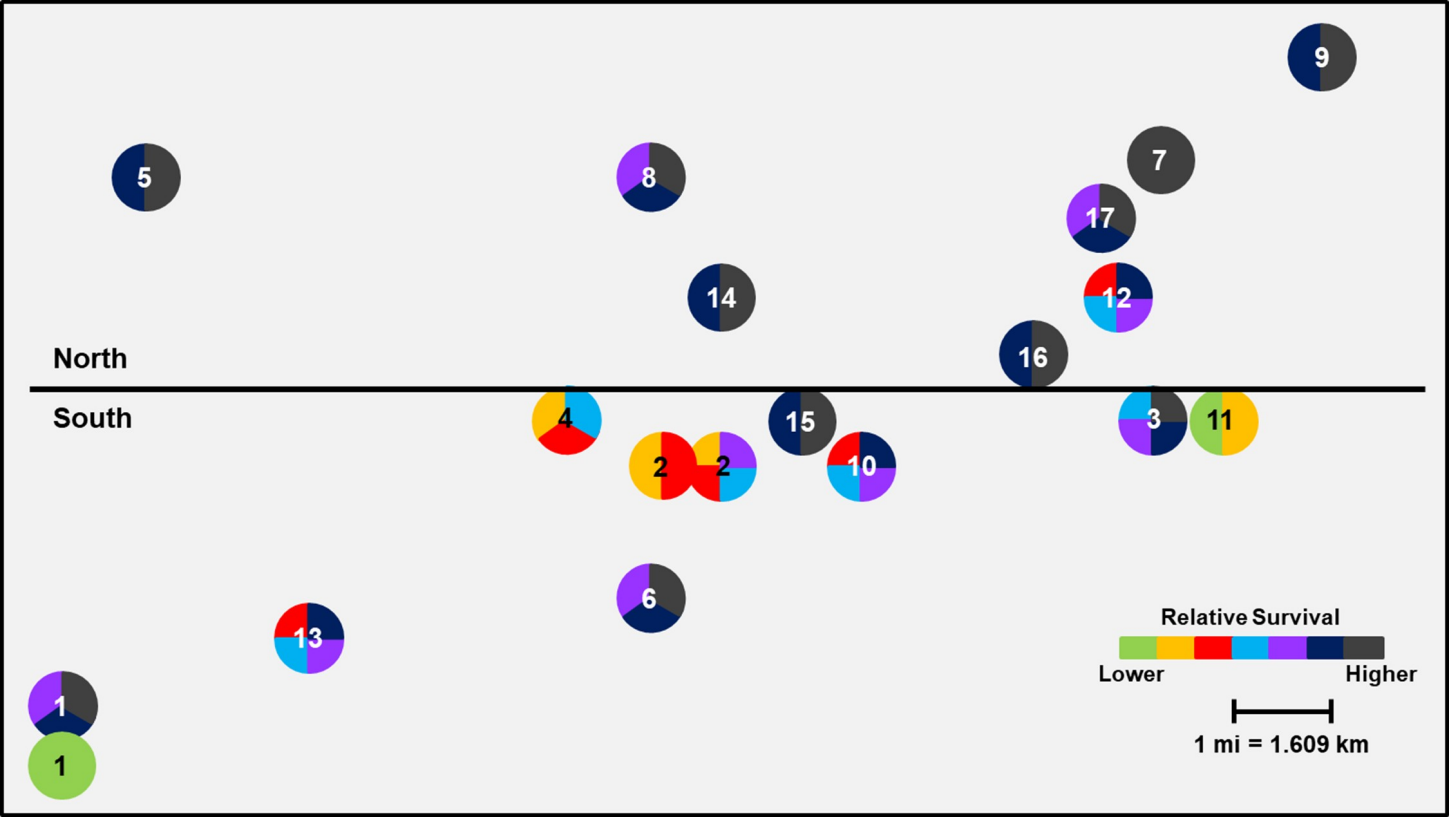
Spatial variation in mean mCry3A survival of WCR larvae among sampling sites in Keith County. Mean proportional survival ranged from 0.05 ± 0.01 to 0.69 ± 0.05. Circles containing the same color(s) are not significantly different (generalized linear model, P > 0.05; LSMEANS option).

The effect of geographic location of larger scale population clusters was highly significant for both Cry3Bb1 (F = 74.42; df = 1, 226; *P*<0.0001) and mCry3A (F = 84.14; df = 1, 226; *P*<0.0001) traits within the Keith County grid. Mean WCR survivorship on Cry3Bb1 in field sites on the south side of the grid (0.160 ± 0.02) was significantly lower than survival in field sites on the north side of the grid (0.394 ± 0.02) (Fig 6). A similar trend was documented with WCR survival on mCry3A (mean survival: south: 0.264 ± 0.02 versus north: 0.541 ± 0.02) (Fig 7). Analyses of small-scale field to field variation in survival excluding the control populations resulted in significant differences among populations in the grids for both Cry3Bb1 (F = 10.96; df = 18, 209; *P*<0.0001) and mCry3A (F = 13.79; df = 18, 209; *P*<0.0001) traits. When mapped, geographically small-scale spatial variation in WCR susceptibility was apparent (Figs 6 and 7).

#### Buffalo County grid

**Fig 8:**
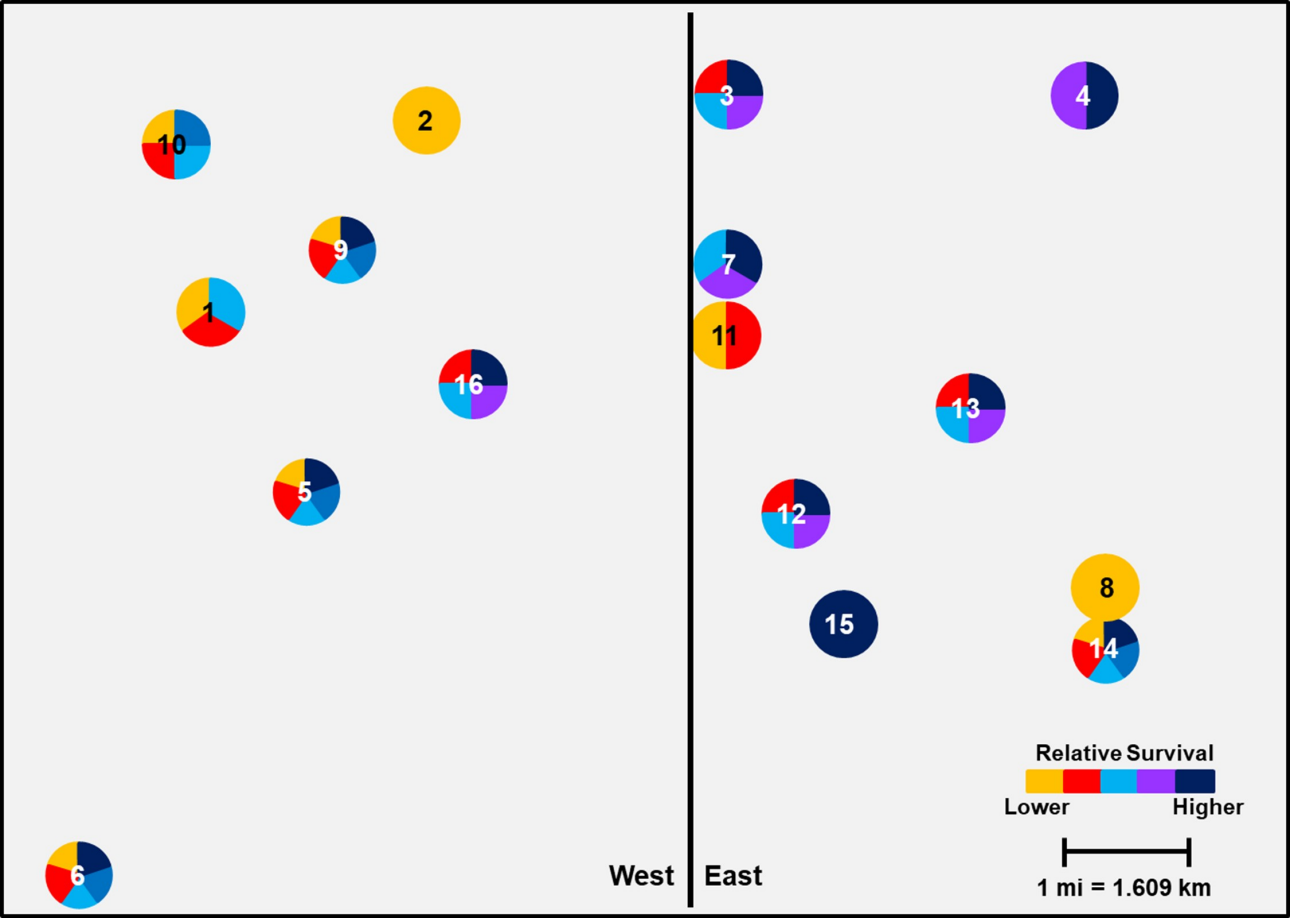
Spatial variation in mean Cry3Bb1 survival of WCR larvae among sampling sites in Buffalo County. Mean proportional survival ranged from 0.07 ± 0.02 to 0.40 ± 0.05. Circles containing the same color(s) are not significantly different (generalized linear model, P > 0.05; LSMEANS option).

**Fig 9:**
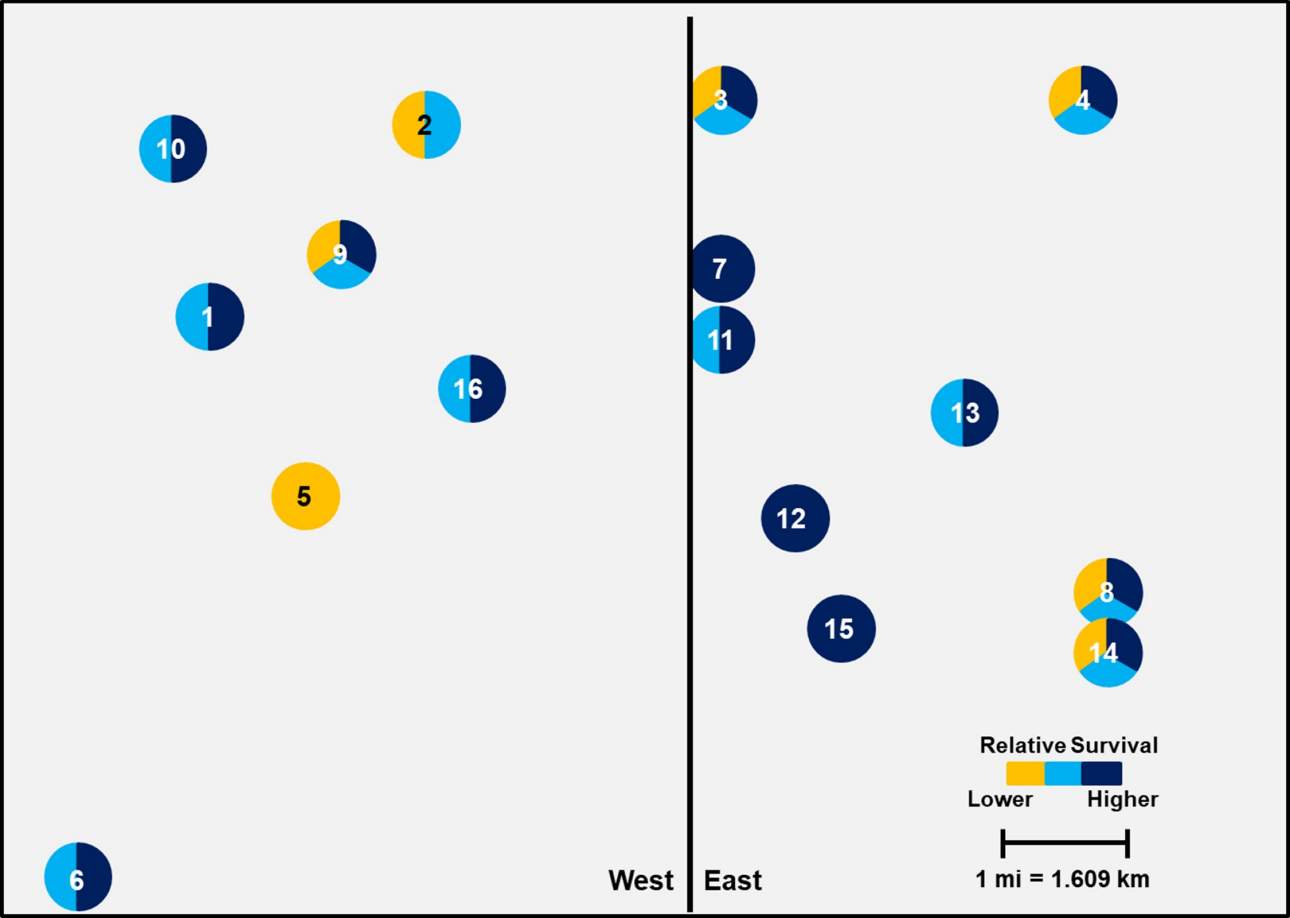
Spatial variation in mean mCry3A survival of WCR larvae among sampling sites in Buffalo County. Mean proportional survival ranged from 0.11 ± 0.03 to 0.46 ± 0.05. Circles containing the same color(s) are not significantly different (generalized linear model, P > 0.05; LSMEANS option).

The effect of geographic location of larger scale population clusters led to a significant difference in mean proportional survivorship from west to east for both Cry3Bb1 (F = 9.29; df = 1, 182; *P* = 0.0026) and mCry3A (F = 9.17; df = 1, 182; *P* = 0.0028). Fields located on the west side of the grid exhibited lower mean WCR survivorship on Cry3Bb1 (0.184 ± 0.02) and mCry3A (0.269 ± 0.02) compared to fields on the east side of the grid (0.260 ± 0.02 and 0.350 ± 0.02 on Cry3Bb1 and mCry3A, respectively) (Figs 8 and 9). Similar to Keith County results, mean proportional survival was significantly different among populations within the grid for both Cry3Bb1 (F = 6.24; df = 15, 168; *P*<0.0001) and mCry3A (F = 4.01; df = 15, 168; *P*<0.0001). When plotted geographically, significant small-scale spatial variation in survival within the sampling grid was again apparent (Figs 8 and 9).

### Evaluation of field history to proportional survival relationship as a predictive tool

**Fig 10:**
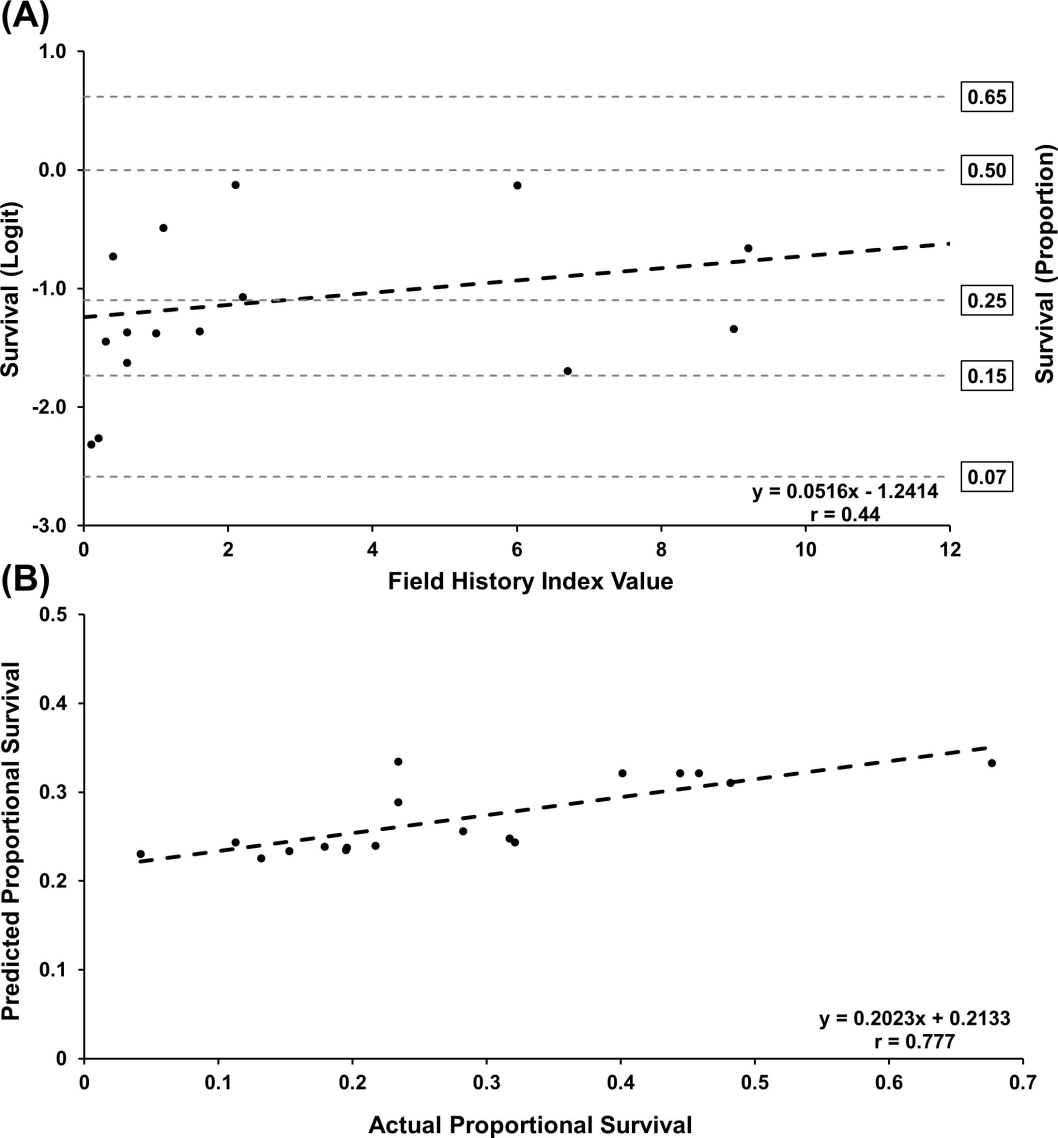
Prediction of 2017 larval survivorship data from field history index values. (A) 2016 regression model used in scoring analysis to predict 2017 survivorship data based on known field history index values. (B) Linear regression analysis of predicted versus actual 2017 survivorship values. Values presented are back-transformed using the ILINK option.

Analysis of the 2016 dataset produced a moderate positive linear relationship between proportional survival and field history index value (r = 0.44) (Fig 10A). This linear model (survival = -1.2414 + 0.05161*field history index value) was used to predict 2017 mean survivorship values, and predicted values were plotted against actual 2017 values resulting in a significant linear regression (survival = 0.2133 + 0.2023*field history index value) explaining approximately 60% of the variation in predicted values (Fig 10B). Correlation analyses indicated a strong positive relationship between predicted and actual survivorship values (r = 0.777, *P* = 0.0001).

## Discussion

The frequency of individuals surviving Cry3Bb1 and mCry3A exposure within Keith and Buffalo county grids consistently ranged from low to very high within years. Incomplete resistance [64] was documented in many populations where mean survival on Bt was significantly greater than survival on the lab control and mean survival on the isoline was significantly greater than survival on the Bt hybrid (Figs 2–5). A few WCR populations also exhibited complete resistance, as defined by Gassmann et al. [43], where mean survival on the isoline and rootworm-Bt hybrid was not significantly different. The occurrence of incomplete resistance, complete resistance, or relative susceptibility among populations in each grid undoubtedly contributed to the significant population*hybrid interaction in each analysis of WCR proportional survival.

When viewed spatially, proportional survival data document a mosaic of local WCR susceptibility to both Cry3Bb1 and mCry3A toxins within the landscape. For example, within the Keith County grid, a high proportion of individuals from field 16 survived exposure to Cry3Bb1 (Fig 2B). Hybrids expressing Cry3Bb1 and mCry3A were frequently planted in this field (Table 1), placing continued selection pressure on this WCR population. In contrast, Cry3Bb1 survivorship of WCR larvae from surrounding fields (3, 11, and 12) was significantly lower compared to field 16 (Fig 6). A similar scenario was evident in Buffalo County when comparing field 15 (high WCR Cry3Bb1 survival, mCry3A history) to fields 8, 12, and 14 (lower WCR survival on Cry3Bb1) (Figs 4 and 8). Thus in both grids, the susceptibility of WCR populations to either Cry3Bb1 or mCry3A in close proximity could be very different.

A number of factors probably contributed to the observed spatial mosaic of WCR susceptibility. WCR larvae from most field sites without previous Bt trait exposure exhibited significantly greater survivorship on Cry3Bb1 (Keith County fields 1, 2, 10, and 11; Buffalo County fields 1, 2, 3, and 5) (Figs 2 and 4 and Tables 1 and 3) and mCry3A (Keith County field 1 [2016]) (Fig 3) expressing hybrids than the lab control. This increased survivorship in fields planted with non-rootworm-Bt hybrids or single-trait Cry34/35Ab1 hybrids indirectly indicates some gene flow of resistant alleles into these fields occurred as a result of beetle movement. However, the large differences in WCR susceptibility observed in some fields in close proximity indicates beetle movement from highly resistant sites did not cause resistant alleles to inundate each grid. This result and the fact that WCR from two fields without trait history (Keith County fields 1 [2016] and 3) (Fig 2) exhibited susceptibility levels that were not significantly different than lab controls collectively suggest that individual fields can be managed within the landscape to slow the evolution of resistance.

The small-scale survival data coupled with known trait histories do provide some examples where possible local adult movement and associated gene flow can be inferred to have affected WCR susceptibility in individual fields. Cry3Bb1- and mCry3A-expressing hybrids were planted in Keith County field 6 consecutively from 2010 to 2016 (Table 1). However, the relative susceptibility of WCR to Cry3Bb1 in the field 6 population was greater than expected based on trait history. This suggests that gene flow of susceptible alleles from surrounding fields (field 2 to the north, south unknown, Fig 2A) may have slowed the evolution of resistance in field 6. Field 2, and two adjacent pivots (not sampled) located one mile north of field 6, each with high WCR densities, had never been planted with Cry3Bb1- or mCry3A-expressing hybrids.

WCR beetle movement enables rapid recolonization of fields after crop rotation or adult management strategies are implemented if a source population is nearby [17,19,55]. Therefore, beetle movement into first-year cornfields could have two significant impacts: 1) in areas with field-evolved resistance, dispersal could significantly negate the impact of crop rotation to slow or mitigate resistance evolution; or 2) dispersal of primarily susceptible individuals into a neighboring field could reduce the proportion of resistant alleles present, slowing resistance evolution. Recolonization after crop rotation was documented in the Buffalo County grid, specifically in field 4. This field was rotated to soybeans in 2011 and 2014 in an attempt to mitigate resistance to Cry3Bb1 (Table 3). However, continuous Cry3Bb1 use in the area directly adjacent to field 4 but outside the sampled grid area allowed recolonization to occur quickly. Many individuals from field 4 survived exposure to Cry3Bb1 in 2016 laboratory bioassays, indicating a high frequency of resistant alleles were present in the immigrant population (Fig 4A). The survival rate in field 4 was not expected based on recent rootworm management history.

Another example of inconsistency between rootworm management history and WCR survival was in Keith County field 1. One of only two fields sampled in consecutive years, the field history indices were low each year (Table 1) but corresponding survival was only low for both Cry3Bb1 and mCry3A during one year (Figs 2 and 3). This was a large field (64.75 ha) and WCR collections were made at different edges of the field each year so it is possible that immigration from neighboring fields may have caused differences in WCR susceptibility along the field edges. It is not unprecedented to find differences in WCR Bt susceptibility across a large field in successive years [49]. This field was at the southwest corner of the Keith County sampling grid where little knowledge of surrounding field histories was available, so a definitive cause and effect is unknown.

Rootworm densities in each field may have been another factor contributing to the small-scale differences in WCR susceptibility in some parts of each grid. Within the Nebraska agroecosystem studied, large monocultures of continuous corn under center pivot irrigation facilitate the buildup of WCR densities over time, potentially affecting gene flow in a positive or negative way. Large densities of a resistant population in close proximity to large densities of a relatively susceptible population may make it difficult for gene flow from either field to quickly alter the resistance allele frequency in each field. Immigrant gene flow may get swamped out by a large resident allele frequency. A possible example of this could have been Keith County field 16 versus fields 3 and 11, which were within 1.5–2.5 kms of each other (Fig 6). Field 16 was highly resistant to Cry3Bb1 and had high WCR densities (Fig 2). In contrast, fields 3 and 11 had never been planted with Cry3Bb1- or mCry3A-expressing hybrids and WCR were ineffectively managed with insecticides, leading to large annual WCR densities. Because much WCR dispersal is trivial [56,57] and many WCR probably remain in their natal field [15,17] in large continuous cornfields, large densities may change the effect of immigration on rate of response to selection pressure that is present in each field.

Cross-resistance was documented between Cry3Bb1 and mCry3A in all WCR populations. However, the ratio of cross-resistance between the two traits was not always 1:1. Variation in cross-resistance has been previously documented in recent literature [42,43]. Gassmann et al. [43] proposed that multiple mechanisms of resistance to Cry3Bb1 potentially exist, which may lead to different levels of cross-resistance to mCry3A. Therefore, it is possible that uneven cross-resistance also contributed to significant variation in susceptibility to Cry3Bb1 and mCry3A.

Even though significant field to field variation in WCR susceptibility to Cry3Bb1 and mCry3A was common throughout the grids, larger scale spatial trends were evident in different sections of each grid. The statistical differences in mean WCR survival when comparing north versus south and east versus west field clusters in Keith and Buffalo County grids, respectively, correlate well with patterns of trait use obtained from field histories. This suggests that greater market penetration and use of Cry3Bb1- or mCry3A-expressing hybrids in parts of each grid (Keith: north; Buffalo: east) over time may have facilitated selection for a higher frequency of resistant individuals in many fields, collectively leading to a patchy neighborhood resistance problem.

In spite of limited sample sizes per year, the base model developed from the field history-2016 bioassay survival relationship (Fig 10A) was effective as a tool to predict 2017 bioassay survivorship. The strong positive correlation between 2017 actual and predicted survivorship values reinforced the importance of individual field history and suggests that management practices applied at the local level are a key driver of selection pressure and evolution of resistance in this system. The importance of this was clearly demonstrated by the sensitivity analyses (data in S4 Table). Length of trait use and associated selection pressure per field were the key variables affecting WCR survivorship in bioassays. Larger sample sizes and more complete knowledge of the system (e.g. surrounding area dynamics/history, WCR densities, other management tactics such as adult insecticide use, etc.) may increase the accuracy of the model.

It was beyond the scope of this study to determine how bioassay results would relate to efficacy of Cry3Bb1- or mCry3A-expressing hybrids in the field. However, mean proportionate WCR survival in a small to moderate number of fields was not significantly different than the field control in bioassays each year (Figs 2–5). This suggests that many of these populations exhibiting low mean survival and incomplete resistance (in relation to the lab control) would still provide adequate control in the field. Cry3Bb1 and mCry3A have provided excellent control in small-plot Bt trait trials conducted at the field control site even though a small shift in susceptibility has occurred over time. In most cases, fields where greater than one approach to WCR management had been used over time, proportional survival in the bioassay was reduced. This provides support for use of an IPM approach to slow the evolution of resistance or mitigate existing low levels of resistance [27,65]. This study provides an empirical demonstration that integrated resistance management and integrated pest management can be complementary and both should be considered together when designing a management program.

In summary, this study documents significant variation in WCR susceptibility to Cry3Bb1 and mCry3A on different spatial scales within two different sampling grids in Nebraska counties. At the local level, results revealed that cornfields in close proximity may support WCR populations with very different susceptibility levels. Comparison of WCR susceptibility in fields without Cry3Bb1 or mCry3A history to fields with trait histories and the lab control indirectly documented that gene flow of resistant alleles occurred in each grid. The impact of gene flow on local susceptibility appeared to vary with individual fields/populations. Based on bioassay survival and field history analyses, management tactics and associated selection pressure at the local level were the key drivers of WCR susceptibility to Bt traits in these grids. With refinement, the field history index concept may be useful to farmers and other ag-professionals as an educational and diagnostic tool to evaluate if an individual field has a heightened risk for WCR resistance evolution. Individual fields and larger-scale field clusters in this study can be viewed as retrospective case histories that can inform development of optimal resistance management programs and increase understanding of plant-insect interactions that may occur when transgenic corn is deployed in the landscape. Results from this study reinforce the current resistance management assumption that resistance evolution begins at the local level [31,41,66] and support current recommendations to slow the evolution of resistance or mitigate existing resistance by using a multi-tactic approach to manage WCR densities in individual fields within an IPM framework [27,67].

## Supporting information

S1 Table Derivation of the field history index values for individual fields. (A) Keith County and (B) Buffalo County.
(PDF)

S2 Table Mean proportional survival (± SE) of lab control populations. (A) Cry3Bb1 in 2016 bioassays, (B) Cry3Bb1 in 2017 bioassays, (C) mCry3A in 2016 bioassays, and (D) mCry3A in 2017 bioassays. Within traits and years, no significant differences in mean survival among populations were documented (generalized linear model, P > 0.05; LSMEANS option).
(PDF)

S3 Table **Mean proportional survival (± SE) of (A) lab and (B) field control populations.** Within a given hybrid, no significant differences in mean survival between years were documented (generalized linear model, P > 0.05; LSMEANS option).
(PDF)

S4 Table Different field history index adjustments used in sensitivity analyses and correlation between actual and predicted survival. (PDF)
