# Supplementary material for: Spatial variation in western corn rootworm (Coleoptera: Chrysomelidae) susceptibility to Cry3 toxins in Nebraska
Source: PLoS One. 2018 Nov 29;13(11):e0208266. doi: 10.1371/journal.pone.0208266 (PMC6264490; doi:10.1371/journal.pone.0208266)
Supplement: S1 Table — (A) Keith County and (B) Buffalo County. (PDF) [file pone.0208266.s001.pdf]

S1 Table. Derivation of the field history index values for individual fields. (A) Keith County and (B) Buffalo County.

(A)

**Year Collected**	**Field Number**	**# years non-Cry3 hybrid**	**# years single-trait Cry3 hybrid**	**# years pyramided Cry3 hybrid**	**3-year selection value**	**Additional years of Cry3 selection?[a]**	**Rotation to non-host crop?**	**Values Negated by Rotation**	**Area Effect**	**Field History Index Value**
**2015**	1	6 x 0.1 = **0.6**	0	0	0	0	No	0	0	0.6
	2	6 x 0.1 = **0.6**	0	0	0	0	No	0	**1.0**	1.6
	3	6 x 0.1 = **0.6**	0	0	0	0	No	0	**-0.5**	0.1
	4	0	2 x 1.0 = **2.0**	2 x 0.5 = **1.0**	0	0	Yes	**Subtract 2.0**	0	1.0
	5	1 x 0.1 = **0.1**	2 x 1.0 = **2.0**	0	0	0	No	0	0	2.1
	6	0	4 x 1.0 = **4.0**	2 x 0.5 = **1.0**	**3.0**	1 x 1.0 = **1.0**<br>2 x 0.5 = **1.0**	No	0	**-1.0**	9.0
	7	2 x 0.1 = **0.2**	2 x 1.0 = **2.0**	1 x 0.5 = **0.5**	**3.0**	0	Yes	**Subtract 0.0**	**1.0**	6.7
	8	2 x 0.1 = **0.2**	4 x 1.0 = **4.0**	0	**3.0**	1 x 1.0 = **1.0**	No	0	**1.0**	9.2
	9	0	3 x 1.0 = **3.0**	0	**3.0**	0	No	0	0	6.0
**2016**	1	7 x 0.1 = **0.7**	0	0	0	0	No	0	0	0.7
	2	7 x 0.1 = **0.7**	0	0	0	0	No	0	**1.0**	1.7
	10	7 x 0.1 = **0.7**	0	0	0	0	No	0	**0.5**	1.2
	11	7 x 0.1 = **0.7**	0	0	0	0	No	0	**-0.5**	0.2
	12	1 x 0.1 = **0.1**	2 x 1.0 = **2.0**	3 x 0.5 = **1.5**	**3.0**	1 x 0.5 = **0.5**	Yes	**Subtract 1.0**	**0.5**	6.6
	13	0	4 x 1.0 = **4.0**	3 x 0.5 = **1.5**	**3.0**	1 x 1.0 = **1.0**<br>3 x 0.5 = **1.5**	No	0	0	11.0
	14	1 x 0.1 = **0.1**	4 x 1.0 = **4.0**	2 x 0.5 = **1.0**	**3.0**	1 x 1.0 = **1.0**	No	0	**0.5**	9.6
	15	1 x 0.1 = **0.1**	3 x 1.0 = **3.0**	3 x 0.5 = **1.5**	**3.0**	1 x 1.0 = **1.0**<br>2 x 0.5 = **1.0**	No	0	**-1.0**	8.6
	16	1 x 0.1 = **0.1**	4 x 1.0 = 4.**0**	2 x 0.5 = **1.0**	**3.0**	1 x 1.0 = **1.0**<br>2 x 0.5 = **1.0**	No	0	**0.5**	10.6
	17	1 x 0.1 = **0.1**	3 x 1.0 = **3.0**	3 x 0.5 = **1.5**	**3.0**	2 x 0.5 = **1.0**	No	0	**1.0**	9.6

[a] Two different values are present in some fields due to additional selection with single-trait and pyramided Cry3 hybrids

(B)

Year Collected	Field Number	# years non-Cry3 hybrid	# years single-trait Cry3 hybrid	# years pyramided Cry3 hybrid	3-year selection value?	Additional years of Cry3 selection?	Rotation to non-host crop?	Values Negated by Rotation	Area Effect	Field History Index Value
**2015**	1	6 x 0.1 = **0.6**	0	0	0	0	No	0	0	0.6
	2	5 x 0.1 = **0.5**	0	0	0	0	Yes	**Subtract 0.3**	0	0.2
	3	4 x 0.1 = **0.4**	0	0	0	0	Yes	**Subtract 0.0**	0	0.4
	4	2 x 0.1 = **0.2**	2 x 1.0 = **2.0**	0	0	0	Yes	**Subtract 2.1**	**1.0**	1.1
	5	5 x 0.1 = **0.5**	0	0	0	0	Yes	**Subtract 0.2**	0	0.3
	6	3 x 0.1 = **0.3**	2 x 1.0 = **2.0**	0	0	0	Yes	**Subtract 0.1**	0	2.2
	7[a]	X	X	X	X	X	X	X	X	X
	8[a]	X	X	X	X	X	X	X	X	X
**2016**	9	6 x 0.1 = **0.6**	1 x 1.0 = **1.0**	0	0	0	No	0	0	1.6
	10	2 x 0.1 = **0.2**	2 x 1.0 = **2.0**	0	0	0	Yes	**Subtract 1.1**	0	1.1
	11	4 x 0.1 = **0.4**	2 x 1.0 = **2.0**	0	0	0	Yes	**Subtract 0.3**	0	2.1
	12	3 x 0.1 = **0.3**	2 x 1.0 = **2.0**	1 x 0.5 = **0.5**	0	0	Yes	**Subtract 1.2**	**1.0**	2.6
	13	1 x 0.1 = **0.1**	3 x 1.0 = **3.0**	0	0	0	Yes	**Subtract 1.0**	0	2.1
	14	5 x 0.1 = **0.5**	0	2 x 0.5 = **1.0**	0	0	No	0	0	1.5
	15	1 x 0.1 = **0.1**	5 x 1.0 = **5.0**	1 x 0.5 = **0.5**	**3.0**	1 x 1.0 = **1.0**	No	0	0	9.6
	16	4 x 0.1 = **0.4**	3 x 1.0 = **3.0**	0	0	0	No	0	0	3.4

[a] Field history data was not available for fields 7 and 8
