# Supplementary material for: Spatial variation in western corn rootworm (Coleoptera: Chrysomelidae) susceptibility to Cry3 toxins in Nebraska
Source: PLoS One. 2018 Nov 29;13(11):e0208266. doi: 10.1371/journal.pone.0208266 (PMC6264490; doi:10.1371/journal.pone.0208266)
Supplement: S2 Table — (A) Cry3Bb1 in 2016 bioassays, (B) Cry3Bb1 in 2017 bioassays, (C) mCry3A in 2016 bioassays, and (D) mCry3A in 2017 bioassays. Within traits and years, no significant differences in mean survival among populations were documented (generalized linear model, P > 0.05; LSMEANS option). (PDF) [file pone.0208266.s002.pdf]

S2 Table. Mean proportional survival (± SE) of lab control populations. (A) Cry3Bb1 in 2016 bioassays, (B) Cry3Bb1 in 2017 bioassays, (C) mCry3A in 2016 bioassays, and (D) mCry3A in 2017 bioassays. Within traits and years, no significant differences in mean survival among populations were documented (generalized linear model, P > 0.05; LSMEANS option).

(A)

Population	Mean Proportional Survival (± SE)
Kansas (KS)	0.025 ± 0.01
Penn I (PA)	0.020 ± 0.009
Whitlock (SD)	0.028 ± 0.01
Linwood (NE)	0.035 ± 0.01

* F=0.66; df=3. 44; *P* =0.5785

(B)

Population	Mean Proportional Survival (± SE)
Kansas (KS)	0.013 ± 0.005
Penn I (PA)	0.015 ± 0.006
Whitlock (SD)	0.015 ± 0.006
Linwood (NE)	0.019 ± 0.007

* F=0.38; df=3,44; *P* =0.7652

(C)

Population	Mean Proportional Survival (± SE)
Kansas (KS)	0.027 ± 0.01
Penn I (PA)	0.038 ± 0.01
Whitlock (SD)	0.050 ± 0.02
Linwood (NE)	0.043 ± 0.02

* F=0.86; df=3,44; *P* =0.4693

(D)

Population	Mean Proportional Survival (± SE)
Kansas (KS)	0.033 ± 0.01
Penn I (PA)	0.043 ± 0.01
Whitlock (SD)	0.024 ± 0.009
Linwood (NE)	0.051 ± 0.02

* F=1.43; df=3,44; *P* =0.2461
