# Supplementary material for: Spatial variation in western corn rootworm (Coleoptera: Chrysomelidae) susceptibility to Cry3 toxins in Nebraska
Source: PLoS One. 2018 Nov 29;13(11):e0208266. doi: 10.1371/journal.pone.0208266 (PMC6264490; doi:10.1371/journal.pone.0208266)
Supplement: S3 Table — Mean proportional survival (± SE) of (A) lab and (B) field control populations. Within a given hybrid, no significant differences in mean survival between years were documented (generalized linear model, P > 0.05; LSMEANS option). (PDF) [file pone.0208266.s003.pdf]

S3 Table. Mean proportional survival (± SE) of (A) lab and (B) field control populations between bioassay years. Within a given hybrid, no significant differences in mean survival between years were documented (generalized linear model, $P > 0.05$; LSMEANS option).

(A)

Hybrid	Mean 2016 Survival ± SE	Mean 2017 Survival ± SE	P-value
Stone 6021RR2	0.565 ± 0.04	0.499 ± 0.04	0.1891
Stone 6021 VT3	0.023 ± 0.01	0.020 ± 0.01	0.4274
Syngenta N68B-GT	0.505 ± 0.03	0.496 ± 0.03	0.8317
Syngenta N68B-3000GT	0.038 ± 0.01	0.040 ± 0.01	0.7960

(B)

Hybrid	Mean 2016 Survival ± SE	Mean 2017 Survival ± SE	P-value
Stone 6021RR2	0.663 ± 0.06	0.578 ± 0.07	0.3683
Stone 6021 VT3	0.113 ± 0.04	0.098 ± 0.05	0.7003
Syngenta N68B-GT	0.513 ± 0.05	0.550 ± 0.05	0.6214
Syngenta N68B-3000GT	0.196 ± 0.02	0.227 ± 0.02	0.2659
