# Supplementary material for: Spatial variation in western corn rootworm (Coleoptera: Chrysomelidae) susceptibility to Cry3 toxins in Nebraska
Source: PLoS One. 2018 Nov 29;13(11):e0208266. doi: 10.1371/journal.pone.0208266 (PMC6264490; doi:10.1371/journal.pone.0208266)
Supplement: S4 Table — (PDF) [file pone.0208266.s004.pdf]

**S4 Table. Different field history index adjustments used in sensitivity analyses and correlation between actual and predicted survival.**

| Field History Index Adjustment | Correlation Coefficient* (p-value) |
|---|---|
| Original Field History Index (base model) | 0.77706 (0.0001) |
| Removal of yearly hybrid values | 0.73852 (0.0005) |
| Removal of selection values | 0.79588 (0.0001) |
| Removal of crop rotation | 0.77893 (0.0001) |
| Removal of area effect | 0.75857 (0.0003) |
| Removal of crop rotation and area effect | 0.76165 (0.0002) |
| Removal of yearly hybrid values, selection values, and crop rotation | 0.18022 (0.4742) |
| Removal of yearly hybrid values, crop rotation, and area effect | 0.71304 (0.0009) |
| Removal of selection values, crop rotation, and area effect | 0.77296 (0.0002) |
| Increase 3-year selection value to 6.0 | 0.76329 (0.0002) |
| Reduce 3-year selection value to 1.0 | 0.78772 (0.0001) |
| Increase area effect by 3x | 0.77893 (0.0001) |

*Represents the correlation between actual and predicted 2017 survivorship values per index adjustment and the significance of the correlation
